# Safety of house dust mite subcutaneous immunotherapy in preschool children with respiratory allergic diseases

**DOI:** 10.1186/s13052-021-01046-z

**Published:** 2021-04-23

**Authors:** Yaqi Yang, Dongxia Ma, Nan Huang, Wenjing Li, Qing Jiang, Yin Wang, Xiaolong Wang, Lin Yang, Rongfei Zhu

**Affiliations:** Department of Allergy, Tongji Hospital, Tongji Medical College, Huazhong University of Science and Technology (HUST), Wuhan, 430030 Hubei China

**Keywords:** Subcutaneous immunotherapy, House dust mite, Allergic rhinitis, Asthma, Side effect

## Abstract

**Background:**

Allergen immunotherapy (AIT) is the only causal therapy for IgE-mediated allergy. There is less evidence about the safety and efficacy of AIT especially subcutaneous immunotherapy (SCIT) in children under 5 years old. We aimed to investigate the side effects and associated risk factors of house dust mite (HDM) SCIT in preschool children with respiratory allergic diseases.

**Methods:**

The preschool children who had HDM-related allergic rhinitis with/without asthma were enrolled and undergone standardized HDM SCIT in our department from June 2013 to December 2019. Local reactions (LRs) and systemic reactions (SRs) were recorded and categorized according to World Allergy Organization recommendations. Demographic data and other therapeutic-related parameters were also recorded to investigate potential risk factors for these side effects.

**Results:**

A total of 91 children (60 boys, 65.93%; 31 girls, 34.07%; mean age 4.13 years old) were included in the study. Among the 91 patients, 3109 SCIT injections were recorded, 62/91 (68.13%) experienced 186 immediate LRs, 4 /91(4.40%) experienced 6 delayed LRs, 11/91 (12.09%) children experienced 44 immediate SRs, 21/44 (47.73%) were grade 1 SRs, 21/44 (47.73%) were grade 2, 2/44 (4.55%) were grade 3, no grade 4 or 5 SRs occurred. Furthermore, 1/91 (1.10%) experienced 1 delayed SRs, manifested by urticaria 2 days later after allergen injection. 9/91 (9.89%) experienced 2 or more times SRs. Multivariable logistic regression analysis showed BMI (OR 1.506; 95%CI 1.091 to 2.079; *p* < 0.05) and sIgE against HDM (OR 1.497; 95%CI 1.082 to 2.071; *p* < 0.05) were risk factors for LRs. No variable was found to correlate with SRs (all *p* > 0.05).

**Conclusions:**

HDM subcutaneous immunotherapy is considered to be safe in preschool children with respiratory allergic diseases. Higher BMI and HDM sIgE level in children are risk factors for developing LRs. The incidence of SRs and the rate of severe SRs are low in preschool children.

## Background

Allergen immunotherapy (AIT) can induce immune tolerance to allergens and has a disease-modifying effect for immunoglobulin E (IgE)-mediated allergic diseases [1, 2]. When allergen extracts are administered, immune responses are elicited, including the activation of specific blocking antibodies (eg. IgG4), tolerance-inducing cells (eg. regulatory T and B cells), and mediators (eg.cytokines including IL-10 and TGF-β) [1]. These responses prevent further exacerbation of the allergen-triggered immune response and attenuate the inflammatory response in tissues [1, 3]. AIT also appears to have a long-term clinical efficacy of up to 12 years even after treatment cessation [4]. In addition, AIT prevents the development of asthma and hypersensitivity to novel allergens [5]. As AIT is the only causal treatment for IgE-mediated allergy, the World Allergy Organization (WAO) recommend AIT can be considered as initial treatment and failure of pharmacotherapy is not an essential prerequisite of the use of AIT [6]. For respiratory allergy, AIT may be proposed as an early treatment in the therapeutic strategy [1, 6]. However, in several AIT guidelines [1, 6], the lower age limit for children eligible for this treatment is set at 5 probably for concerns that children under 5 show less cooperation and limited ability to report the early signs and symptoms of severe allergic side-effects in the age. These concerns are understandable but not necessarily well-supported by evidence.

It is well known that natural history or typical progression of allergic diseases (namely allergy march) such as atopic dermatitis, food allergy, allergic rhinitis (AR) and asthma often begin early in life, while AIT is the only treatment that may alter the progression of allergic diseases [7, 8], the decision of initial AIT in age below 5 years old group always depends on how to balance the benefit of early intervention and risk of potential side effects. For safety concerns, children under 5 have been listed in the EAACI guideline as a relative contraindication of AIT, both in sublingual immunotherapy (SLIT) and subcutaneous immunotherapy (SCIT). However, some studies had shown that SLIT in children below 5 years old were effective and there was no difference in the safety profile in this age group and older children. One study included children aged 2–5 years receiving SLIT for house dust mite (HDM) allergy, only mild-to-moderate local adverse reactions were reported [9]. For SCIT, there have been reported a relative higher risk of side effects including local reactions (LRs) and systemic reactions (SRs) than SLIT. In SCIT trials, SRs occurred in 6–17% of pediatric asthma patients [10], grade 1 reactions were the most frequent in both adults and children [11]. There are limited studies of SCIT in under-five age group. A retrospective study of SCIT in 239 children below the age of 5 years (8–59 months old), who received a total of 6689 injections, reported a single systemic reaction 90 min after an injection in a 3-year-old boy [12]. According to current data, adverse effects of AIT were not more frequent or more severe in children below 5 years old group.

HDM is one of the most common sources of indoor allergens and can trigger perennial AR, asthma. Our previous study found that HDMs were the major aeroallergens among AR patients in central China, with a sensitization rate of over 90%. In the confirmed AR children below 6 years old, the sensitization rate of HDM [including *Dermatophagoides farina* (Df) and *Dermatophagoides pteronyssinus* (Dp)] was 97.6% [13]. SCIT with commercial HDM extracts of Dp (Alutard SQ, ALK Hørsholm, Denmark) have been demonstrated to be effective in children with AR and/or asthma [14–16]. This study aimed to investigate the incidence of side effects and potential risk factors during HDM SCIT in preschool children with respiratory allergy.

## Methods

### Design and participants

We conducted a prospective study among preschool children treated by SCIT in the Department of Allergy, Tongji Hospital. Participants included preschool children treated by standardized SCIT with Alutard Dp vaccine, from June 2013 to December 2019. The study was approved by the Independent Ethical Committee of Tongji Hospital, and each participator’s statutory guardian signed the informed consent of the immunotherapy and this study. The inclusion criteria were as follows: (1) aged ≤5 years; (2) diagnosed with AR with or without asthma according to the Allergic Rhinitis and its Impact on Asthma Guidelines(ARIA) [17] and Global Initiative for Asthma (GINA) (https://ginasthma.org/); (3) positive skin-prick tests (a wheal diameter ≥ 3 mm) to Df and Dp (Macro-Union Pharmaceutical, Beijing, China) and serum specific IgE (sIgE) against Df and Dp ≥ 0.7 kU/L (Thermo-Fisher, Uppsala, Sweden); (4) allergic symptoms of AR and/or asthma after exposure to HDM; (5) received at least one dosage HDM vaccine injection. The exclusion criteria were as follows: (1) sensitization and symptoms after exposure to other allergens (sIgE ≥0.7 kU/L) such as pollens and molds, and experiencing symptoms after allergen exposure; (2) presence of autoimmune diseases, primary immunodeficiency diseases, type III allergic diseases and neoplasia.

### Treatment

The treatment regimen was set up according to the conventional schedule provided by the manufacturer (Alutard SQ, ALK Hørsholm, Denmark). During the build-up phase of SCIT, patients received weekly injections at a dose of 0.2, 0.4, and 0.8 mL in No. 1 (100SQ/mL), No.2 (1000SQ/mL) to No.3 vials (10,000SQ/mL) and 0.1, 0.2, 0.4, 0.6, 0.8, and 1.0 mL in No. 4 vial (100,000SQ/mL, contains 9.8 μg *Der p1* per mL) [13] until reaching an optimal dose. Subsequently, the maintenance dose was given every 4–6 weeks for 3–5 years. Before each injection, patients were required to undergo the following: (1) physical examination; (2) peak expiratory flow (PEF) test (measured by a portable mini peak flowmeter, Wanbo Technology Co., Ltd., Shanghai), patients with PEF < 80% of the predicted value (or personal best value) were not allowed to receive allergen injection); (3) pulmonary function testing, recorded forced expiratory volume in 1 s (FEV1) (measured by an EasyOne™ spirometry, NDD Co.,LTD, Switzerland), patients with FEV1 < 80% of the predicted value (or personal best value) were not allowed to receive injection. Both PEF and FEV1 were detected only in children above the age of 4; (4) assessment of side effects (especially delayed local and systemic reactions provided by their parents) since last injection. Patients were kept in the clinic for at least 30 min after each injection. Patients who experienced SRs were treated with rescue medications immediately and monitored carefully. If delayed LRs and/or SRs happened, the parents were asked to report to our center, and the doctors would determine further treatment.

### Combined symptom medication score (CSMS)

We evaluated the efficacy of SCIT by combined symptom medication score (CSMS), which was based on an equal weight of the daily symptom score (dSS) (0–3) and of the daily medication score (dMS) (0–3) in the daily total CSMS (0–6) according to AIT position paper [18]. We recorded the visual analog scale (VAS; a straight line was scaled as 0 to 10 cm, with “0” indicating “no symptoms” and “10” for “most serious symptoms” [18]): parents assessed the nasal symptoms of children in the past 3 days before injection. We converted the mean VAS score (ranged from “0” to “10”) within 180 days into symptom score (ranged from “0” to “3”). For example, if one patient’s VAS scores were “5”, “5”, “4” and “4” during the past 180 days, then the mean VAS score was (5 + 5 + 4 + 4)/4 = 4.5, the symptom score would be 4.5*3/10 = 1.35. As it was impractical to record the daily medication score (dMS) in in this open-labelled non-controlled 3-year prospective study, we calculated the total medication amounts within 180 days and converted that into the average medication dose. The medication scoring criteria were as following:“0” for “no medication”; “1” for “180-day dose of oral nonsedative H1 antihistamines (H1A) (the standard dose was 5mg loratadine daily or equal)”; “2” for “6 bottles of mometasone furoate nasal spray (180 sprays) or equal dose of other nasal steroid spray”, the total medication score was ranged from “0” to “3”. For example, if one patient used 1 bottle of mometasone furoate nasal spray and 120 mg loratadine within 180 days, then the medication score would be [(1/6)*2 + 120/ (180*5)] = 0.46. The CSMS was the sum of symptom score and medication score, ranging from “0” to “6”.

### Side effects

Side effects were documented at the time of each injection, including LR and SR. Erythema and/or swelling at the injection area were defined as large LR (≥4 cm in diameter) [19, 20]. SRs were classified into 5 grades, ranging from grade 1 (symptoms of one organ system present) to grade 5 (death) according to the AIT Systemic Reaction Grading System proposed by WAO [21, 22]. In our study, the side effects were further classified into immediate (appearance within 60 min after injection) and delayed (occurring after 60 min) side effects.

### Statistical analysis

STATA 15.1 (StataCorp, College Station, Texas 77,845 USA) was used for data analysis. All variables were converted into numeral values and analyzed as absolute or relative frequencies, mean and standard deviation (SD) to describe the demographic data, diagnostic, therapeutic parameters and side effects.

2-sample *t* test was used to evaluate the continuous variable. The Pearson chi-square test and Fisher’s exact test were used to determine the association between categorical variables. Odds ratios (ORs) between groups were calculated and 95% confidence intervals (CIs) were generated. For the multivariable analysis, logistic regression, with forwarding model selection and the likelihood ratio test, was applied to assess the predictive model of the dependent variable. All tests were performed 2-tailed, and *p* < 0.05 was considered statistically significant.

## Results

### Study population

In total, 91 preschool patients (60 boys, 65.93%; 31 girls, 34.07%) were included in this study. The mean age was 4.13 ± 0.57 years old. Of the patients, 80 (87.91%) had AR, 11(12.09%) had AR with asthma. The mean number of injections per patient was 34.17 ± 16.00, the mean length of SCIT was 105.50 ± 70.39 weeks (Table 1).

**Table 1:** Demographic data

Characteristics	*n* = 91
Gender	
Male, n (%)	60 (65.93)
Female, n (%)	31 (34.07)
Age (years), mean (SD)	4.13 (0.57)
BMI (kg/m^2), mean (SD)	15.48 (1.64)
Diagnosis	
AR, n(%)	80 (87.91)
AR with asthma, n(%)	11 (12.09)
Family history	
None, n(%)	54 (59.34)
Father, n(%)	23 (25.27)
Mother, n(%)	14 (15.38)
AD in infancy	
Yes, n(%)	53 (58.24)
No, n(%)	38 (41.76)
Food allergy history	
Yes, n(%)	35 (38.46)
No, n(%)	58 (63.74)
Dp sIgE at baseline (KU/L), mean (SD)	50.23 (38.35)
Injections per patient, mean (SD)	34.17 (16.00)
Length of SCIT (weeks), mean (SD)	105.50 (70.39)

*BMI* body mass index, *AR* allergic rhinitis, *AD* Atopic dermatitis, *SD* standard deviation, *Dp* Dermatophagoides pteronyssinus, *sIgE* specific immunoglobulin E, *SCIT* subcutaneous immunotherapy

### Incidence of side effects

In total, 91 preschool patients received 3109 injections, 62 (68.13%) experienced 186 (5.98%) immediate LRs, 11 (12.09%) experienced 44 (1.42%) immediate SRs, 9 (9.89%) experienced ≥2 times SRs, 4 (4.40%) experienced 6 (0.19%) delayed LRs, 2 (2.20%) experienced ≥2 times delayed LRs and relieved in three days without extra medication. 1/91 (1.10%) experienced delayed 1 SR in the build-up period, manifested by mild urticaria 2 days later after allergen injection, the symptoms were relieved in one day after taken oral anti-histamine medication.

### Severity of side effects

Among the 44 immediate SRs, 97.73% (43/44) occurred during No. 4 vial (100,000 SQ-U/mL) injection, 47.73% (21/44) were grade 1 SRs, 47.73% (21/44) were grade 2, 4.55% (2/44) were grade 3, no grade 4 or 5 SRs occurred. The mean reaction dose of SRs was 55,245.45 ± 24,594.38 SQ (approximate 0.55 ml of Vial No.4). The symptoms/signs of the SRs included conjunctival pruritus, rhinitis symptoms, itchy throat, cough not related to bronchospasm, urticaria and generalized pruritus, asthma symptoms/signs (cough, wheezing, shortness of breath), declines in PEF or FEV1 and abdominal cramps (Table 2). The patients with SRs responded rapidly to rescue medications such as oral H1 antihistamines and inhaled β2 agonists. Both patients with grade 3 had treatment with intramuscular epinephrine.

**Table 2:** Manifestations and severity of systemic reactions

	SRs	SR rates (‰ of injections) *N* = 3109
Organ systems involved		
Conjunctival (pruritus)	1	0.32
Upper respiratory (rhinitis, itchy throat or cough not related to bronchospasm)	20	6.43
Cutaneous (urticaria and generalized pruritus)	2	0.64
Lower respiratory (asthma, wheezing rhonchi or drop of PEF or FEV1)	21	6.75
Gastrointestinal (abdominal cramps)	1	0.32
Grade 1	21	6.75
Grade 2	21	6.75
Grade 3	2	0.64

*SR* systemic reaction, *PEF* peak expiratory flow, *FEV1* forced expiratory volume in 1 s

**Fig. 1:**
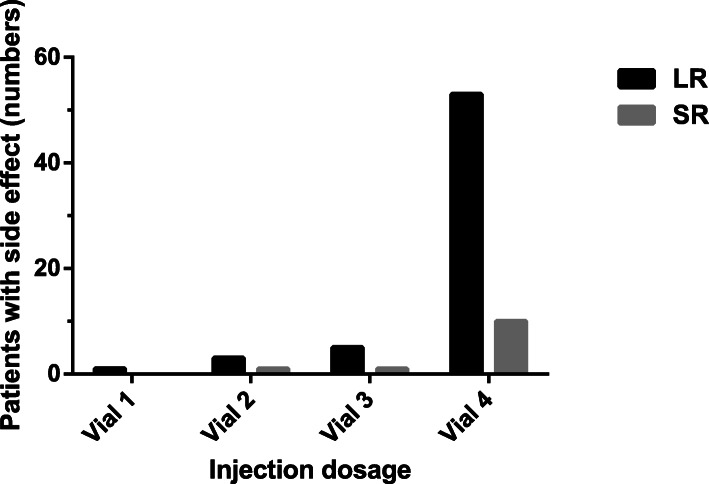
Patients experienced side effects in different dosages

Among the 64 patients with side effects, 82.81% (53/64) experienced first side effect during No.4 vial injection, 87.5% (56/64) had LRs as the first side effects (Fig. 1). In total, 26.37% (24/91) patients couldn’t reach the maximum dosage recommended by the manufacturer (100,000SQ), 75% (18/24) of them were because of large LR, 25.00% (6/24) of them were because of SRs. No patient dropped out due to pain or large LRs.

### Risk factors of LRs and SRs

Nine variables including demographic factors and Dp sIgE level were included in the analysis, only the BMI were found to correlated to LRs (OR 1.419; 95%CI,1.053 to 1.913; *p* < 0.05) (Table 3). However, multivariable logistic regression analysis showed BMI (OR 1.506; 95%CI, 1.091 to 2.079; *p* < 0.05) and Dp sIgE level (OR 1.497; 95%CI, 1.082 to 2.071; *p* < 0.05) were risk factors for LRs.

**Table 3:** Univariable Predictors of LRs

Variable	*p*	OR(95%CI)
Age	0.688	
Gender	0.595	
BMI	0.022*	1.419 (1.053–1.913)
Diagnosis	0.558	
Disease duration	0.596	
AD in fancy	0.159	
Food allergy history	0.943	
Family history	0.328	
Dp sIgE	0.074	

*OR* odds ratio, *CI* confidence interval, *BMI* body mass index, *AD* Atopic dermatitis, *Dp* Dermatophagoides pteronyssinus, *sIgE* specific immunoglobulin Es

*p < 0.05

Ten variables were included in SR analysis, only the LRs showed a correlation to SRs (OR 1.231; 95%CI,1.018 to 1.488; *p* < 0.05) (Table 4). However, in the multivariable logistic regression analysis, no variables were found to have correlation to SRs (all *p* < 0.05).

**Table 4:** Univariable Predictors of SRs

Variable	*p*	OR(95%CI)
Age	0.780	
Gender	0.864	
BMI	0.625	
Diagnosis	0.099	
Disease duration	0.705	
AD in fancy	0.699	
Food allergy history	0.879	
Family history	0.228	
Dp sIgE	0.079	
LR	0.032*	1.231 (1.018–1.488)

*OR* odds ratio, *CI* confidence interval, *BMI* body mass index, *AD* atopic dermatitis, *Dp* Dermatophagoides pteronyssinus, *sIgE* specific immunoglobulin Es, *LR* local reaction

*p < 0.05

### Correlation between side effects and SCIT efficacy

**Fig. 2:**
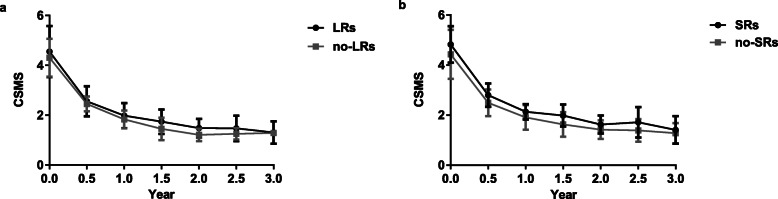
The CSMS in different years. **a** The CSMS were no differences in the patients with LRs and without LRs (*p*>0.05); **b** The CSMS were no differences in the patients with SRs and without SRs (*p*>0.05)

The CSMS showed a decline trend in this population, from 4.47 ± 0.95 at baseline to 1.94 ± 0.47 at year 1, 1.45 ± 0.37 at year 2 and 1.31 ± 0.43 at year 3 (all *p* < 0.05 compared with baseline). However, no correlations were found among the side effects and SCIT efficacy in this population. The CSMS were similar in the patients with LRs and without LRs, as well as the patients with/without SRs (*p* > 0.05) (Fig. 2).

## Discussion

Allergen immunotherapy in preschool children is always a dilemma for the physicians. On the one hand, there are strong evidences to support early introduction of AIT to prevent allergy march [7, 8]; on the other hand, younger children always show stronger resistance to AIT and cannot depict their symptoms accurately when adverse reactions happen. Moreover, they have often upper respiratory infections resembling allergic symptoms. Recently some studies suggested SLIT was rarely associated with moderate or severe SRs and SLIT was considered to be safe in children younger than 5 years old [23, 24]. However, due to the limited studies of SCIT and SLIT in under-five age group, most guidelines have no recommendation of AIT in this population. In our study, we investigated the safety profile of the HDM SCIT in preschool children by examining the incidence of LRs and SRs with conventional SCIT regimen. We found that the HDM SCIT could be considered safe in preschool children with respiratory allergic diseases. In addition, during maintenance phase one subcutaneous injection per month was adequate in order to maintain efficacy of SCIT.

The immediate LRs in preschool patients were common in our study. Nearly 70% of our patients experienced immediate LRs during SCIT. It is easy to understand that the majority of LRs happened in high dosage HDM extracts (No.4 vial) injection. The incidence of immediate LRs in preschool patients was similar to that observed in patients aged 5–60 years in our previous study [15]. This suggests that SCIT in age below 5 years old group is as tolerable as for children aged above 5 and adults. Some reports showed that up to 93% of patients experienced immediate LRs during SCIT [10, 25, 26]. The differences of immediate LRs rates may be due to the allergen type and allergen extracts used in different studies. For example, pollen extracts and depot allergen extracts were easier to evoke LRs than HDM and aqueous allergen extracts [27]. The HDM vaccine we used in our study were Al (OH)3 absorbed sustain-released extracts, which may lead to a relatively high incidence of LRs. Interestingly, the delayed LRs appeared less than those in other studies, it might be related to the knowledge and judgment of the parents, as the delayed LRs were usually recorded in home, sometimes the delayed LRs records were missing. Risk factors analysis showed that children with high specific IgE level and high BMI were prone to develop LRs. We speculate this might be due to that the adipose tissue limited the diffusion of allergen extracts in high BMI children. However, this finding needs to be validated in larger population. Since LRs usually disappeared in a period ranging from several hours to 3 days and didn’t need any medical intervention in most cases, the attempt to reduce LRs by dosage adjustment may not be necessary in this population, unless large LRs happens and the LRs become intolerable.

We found the incidence of immediate and delayed SRs in the preschool patients was similar to that previously reported in children [28–30], 12.09% of the population experienced SRs during SCIT. In some Chinese patients’ studies, SRs occurred in 12.26–18.49% of patients and 0.72–3.28% of injections in children. We also found the majority of the SRs were grade 1 and grade 2 reactions, which was consistent with previous studies [31, 32]. Only 2 /91 (4.55%) patients experienced grade 3 reactions within 30 min after injection and responded well to rescue medication, no patient had grade 4 or grade 5 reaction. These indicated that SCIT in age below 5 years old was as safe as in older children. Indeed, to assure the safety of these patients, we took an extra procedure that all the patients—including AR patients— to finish the PEF test before each injection. One challenge was that the younger children might not get the PEF value accurately. Thus, we didn’t expect the accuracy but require the good repeatability of the data. These mutually-confirmed data obtained from mini-PEF flowmetry and portable spirometry were utilized to ensure the eligibility to receive allergen injection. The preschool patients would postpone their injection if the PEF value didn’t achieve 80% of their predict value (or personal best value), which might be helpful to reduce the incidence of SRs in this population. Despite the extra step we had taken in our study, we still found the incidence of SRs were slightly higher than those in adolescents or adults (0.31–1.47% of injections, 5.68–10.98% of patients) [14, 30]. Another study also showed that the incidence of side effects was higher in the preschool (2 to 6 years old) group than the older (7 to 18 years old) group [33]. Considering that the majority of SRs happened in high dose allergen injection (usually Vial No.4) and the preschool children shared the same treatment regimen with the adolescents and adults, the maximum tolerable dosage in this population needs to be further investigated to make the balance between efficacy and safety. The dose of HDM extracts would be appropriately reduced after SRs if SCIT was continued, we usually reduced the dose to one that was previously tolerated or an even lower dose if the reaction was severe [34].

We found that LRs showed a correlation to SRs, but this correlation was not confirmed in the multivariable logistic regression analysis. Indeed, the correlation between LRs and SRs is still in controversy. Previous studies indicate that individual local reactions do not appear to be predictive of subsequent SRs. However, some patients with a greater frequency of large LRs might be at an increased risk of future SRs [34]. In our study, the children with large LRs would reduce their allergen dosage in the next injection as Cox et al. suggested [34], which might help to decrease the risk of SRs. Asthma (especially moderate asthma) has been identified as a risk factor for SRs in many studies [14, 35, 36]. However, asthma was not a risk factor for SRs in our population, which might be attributed to the fact that all of the 11 asthmatic children have mild asthma, and also the standardized pre-treatment evaluation procedure and careful allergen-dose adjustment during SCIT, as more than 1/4 of our preschool patients didn’t reach the recommended maintenance dosage (100,000 SQ) for adolescents and adults. We also found other variables including age and disease duration had no correlation with SRs. In other words, the SRs sounds to be unpredictable in the preschool patients, we need to be very caution for each injection to recognize the early signs of SRs in time.

Some limitations hampered our study. Firstly, we did not have placebo group (only contain adjuvant) and older-age group serveing as control group. Thus, this study was meant to be regarded as a descriptive research and the safety profile in this study needs to be interpreted with caution. Secondly, it is quite challenge to acquire the accurate value of PEF and FEV1 in these younger-age population. For this reason, we couldn’t present the lung function data in the results as evidence of SCIT efficacy. Instead, we listed this step as a part of pre-injection procedure in the SCIT. Finally, the number of children with asthma (11/91) was small in our study, which might lead to bias and made it difficult to evaluate the correlation between asthma and SCIT-related adverse reactions. More asthma cases are needed in further studies to figure out the relationship between asthma and SCIT safety in the preschool children.

## Conclusions

Our study demonstrates that HDM SCIT is considered to be safe in preschool children with respiratory allergic diseases. The incidence of SRs is low and the severity of SRs ranges from mild to moderate in the preschool patients. LRs and SRs usually happen when high dosage allergen vaccine is administrated. Children with higher BMI and HDM sIgE level are prone to develop LRs. A comprehensive pre-treatment evaluation and careful allergen-dosage adjustment help to decrease the side effects of HDM SCIT in the preschool children.

## Data Availability

All data generated or analyzed during this study are included in this published article.

## Abbreviations

AIT: Allergen immunotherapy
IgE: Immunoglobulin Es
WAO: World Allergy Organization
AR: Allergic rhinitis
SLIT: Sublingual immunotherapy
SCIT: Subcutaneous immunotherapy
HDM: House dust mite
LR: Local reaction
SR: Systemic reaction
Df: Dermatophagoides farinae
Dp: Dermatophagoides pteronyssinus
ARIA: Allergic Rhinitis and its Impact on Asthma Guidelines
GINA: Global Initiative for Asthma
PEF: Peak expiratory flow
FEV1: Forced expiratory volume in 1 s
CSMS: Combined symptom medication score
VAS: Visual analog scale
DSS: Daily symptom score
DMS: Daily medication score
SD: Standard deviation
OR: Odds ratio
CI: Confidence interval
